# Leukemia cutis and anterior uveitis associated with chronic myelomonocytic leukemia

**DOI:** 10.1016/j.jdcr.2024.07.001

**Published:** 2024-07-22

**Authors:** Kamran K. Harper, Vanessa Mutch, Jordan Philip Safran, Daisy Wu, Nidhi Aggarwal, Ekta Maheshwari, Allison K. Weinstock, Kevin F. Gibson, Marie-Helene Errera, Jing-Zhou Hou, Arivarasan D. Karunamurthy, Yuri L. Bunimovich

**Affiliations:** a Department of Dermatology, University of Pittsburgh Medical Center, Pittsburgh, Pennsylvania; b Sidney Kimmel Medical College of Thomas Jefferson University, Philadelphia, Pennsylvania; c Department of Pathology, University of Pittsburgh Medical Center, Pittsburgh, Pennsylvania; d Department of Radiology, University of Pittsburgh Medical Center, Pittsburgh, Pennsylvania; e Division of Pulmonary, Allergy, Critical Care, and Sleep Medicine, Department of Medicine, University of Pittsburgh, Pittsburgh, Pennsylvania; f Department of Ophthalmology, University of Pittsburgh Medical Center, Pittsburgh, Pennsylvania; g Division of Hematology-Oncology, Department of Medicine, Hillman Cancer Center, University of Pittsburgh Medical Center, Pittsburgh, Pennsylvania

**Keywords:** autoimmune, chronic myelomonocytic leukemia, leukemia cutis, myelodysplastic, myeloproliferative, uveitis

## Introduction

Chronic myelomonocytic leukemia (CMML) is an uncommon myeloid neoplasm with myeloproliferative and myelodysplastic features characterized by peripheral monocytosis and cytopenia.^1^ CMML may have cutaneous manifestations known as leukemia cutis (LC).^2^ LC in the setting of CMML may portend malignant transformation to acute myeloid leukemia (AML).^2^ The distribution and morphologic features of LC in CMML are highly variable and often nonspecific.^2^ CMML may coexist with numerous secondary autoimmune and inflammatory processes; this phenomenon presents a clinical challenge which can delay correct diagnosis and appropriate therapeutic management. Thus, careful consideration of extramedullary manifestations of CMML and associated autoimmune conditions in the skin and other organs greatly aids in the correct and timely diagnosis.

## Case report

**Fig 1:**
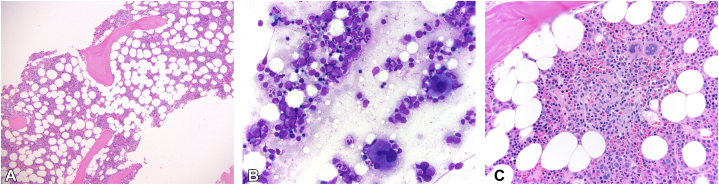
Bone marrow biopsy images: (**A**) H&E stain 10×, (**B**) PAS stain 40×, and (**C**) H&E stain 40×, demonstrating mildly hypercellular marrow (40% to 50%) with trilineage hematopoiesis and scattered non-necrotizing interstitial granulomas without increased blasts, normal myeloid to erythroid ratios with normal, sequential maturation and no dyspoesis, the presence of megakaryocytes, and normal appearing bony trabeculae.

**Fig 2:**
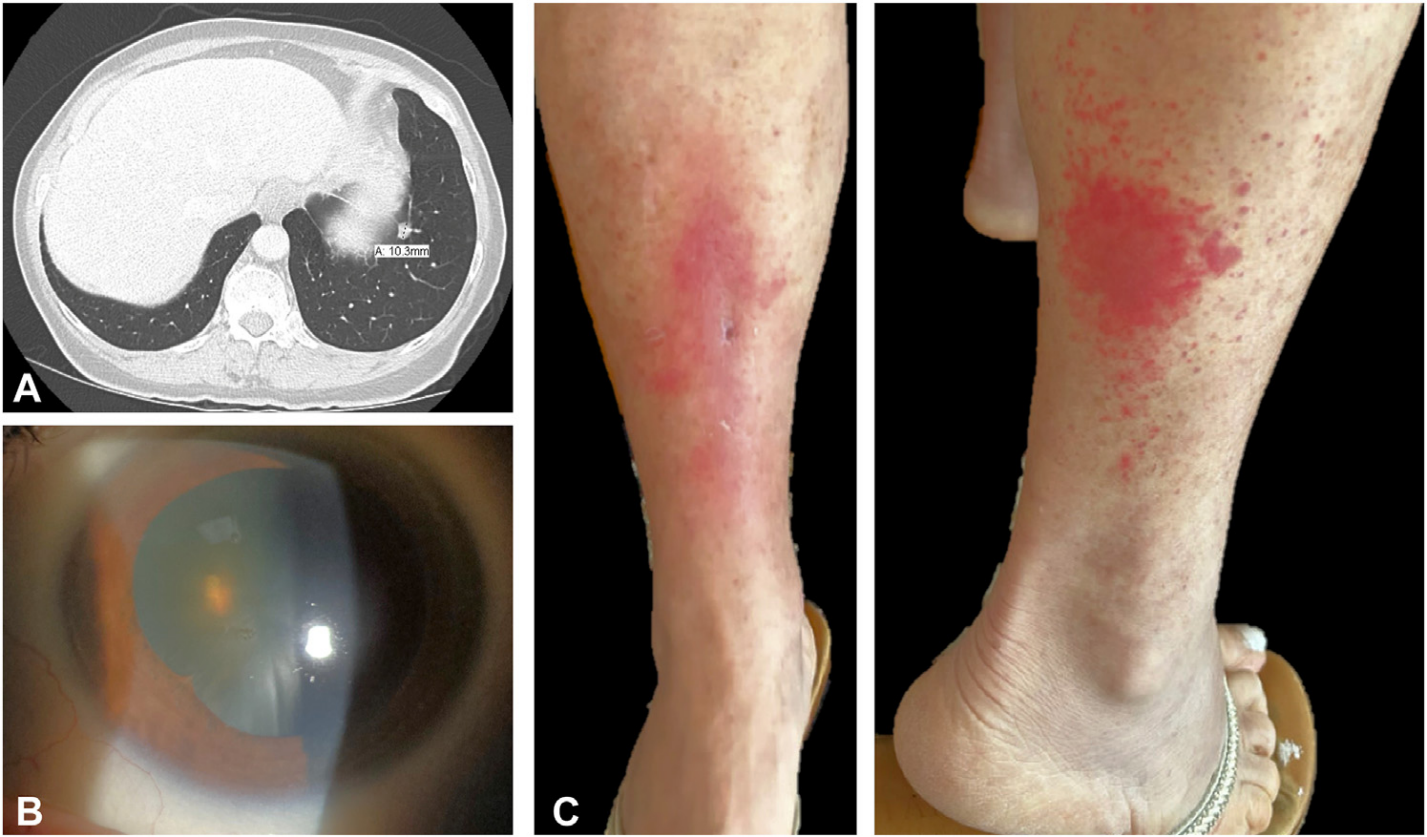
**A,** Computed tomography of the chest showing a 1 cm pulmonary micronodule. **B,** Sequela of anterior uveitis in the form of a focal posterior synechiae in the left eye. **C,** Poorly defined, erythematous non-scaly patch on the anterior leg, and brightly erythematous papules centrally coalescing into a plaque clustered on the posterolateral leg.

A 76-year-old female presented to dermatology with a 4-year history of unilateral and bilateral, relapsing, remitting, painful, lower extremity edema and erythema. Her skin eruptions were repeatedly diagnosed by other specialists as recurrent cellulitis and treated with multiple courses of antibiotics. She also had a diagnosis of myelodysplastic syndrome based on persistent thrombocytopenia and a bone marrow (BM) biopsy from 3 years prior demonstrating hypercellular marrow with trilineage hematopoiesis (40% to 50% cellular) and scattered non-necrotizing interstitial granulomas without increased blasts (Fig 1). Acid-fast bacillus and Grocott stains were negative. At that time, computed tomography of the chest, abdomen, and pelvis revealed multiple pulmonary micronodules, mild splenomegaly, and enlarged peripancreatic, mesenteric, gastrohepatic, paraceliac, retroperitoneal, and iliac lymph nodes (Fig 2, *A*). Several months prior to her presentation to dermatology, the patient was diagnosed with sarcoidosis based on the above history and clinical findings. On ophthalmologic evaluation, the patient was found to have bilateral anterior uveitis which was also initially attributed to sarcoidosis (Fig 2, *B*).

At presentation, the patient endorsed an unintentional 14-pound weight loss, chronic chills, night sweats, and bilateral photophobia with blurry vision. On physical examination, she had well-demarcated erythema and edema of the dorsal right foot extending to the ankle proximally and the metatarsal phalangeal joints distally, with palpable, full, anterior tibial, and dorsalis pedis pulses. The morphology of the patient’s skin eruptions varied between subsequent office visits (Fig 2, *C*). Blood work revealed anti nuclear antibodies of 1:640 (nuclear speckled), hypocomplementemia (C3 22 mg/dL, C4 <8 mg/dL), elevated C-reactive protein (3.810 mg/dL), and positive Sjögren's-syndrome-related antigen. Treatments with hydroxychloroquine, methotrexate, and azathioprine caused immediate exacerbations of her lower extremity rash, edema, pruritus, and pain, along with dyspnea. Her symptoms and cutaneous eruption briefly resolved with a prednisone taper but recurred soon after completion.

**Fig 3:**
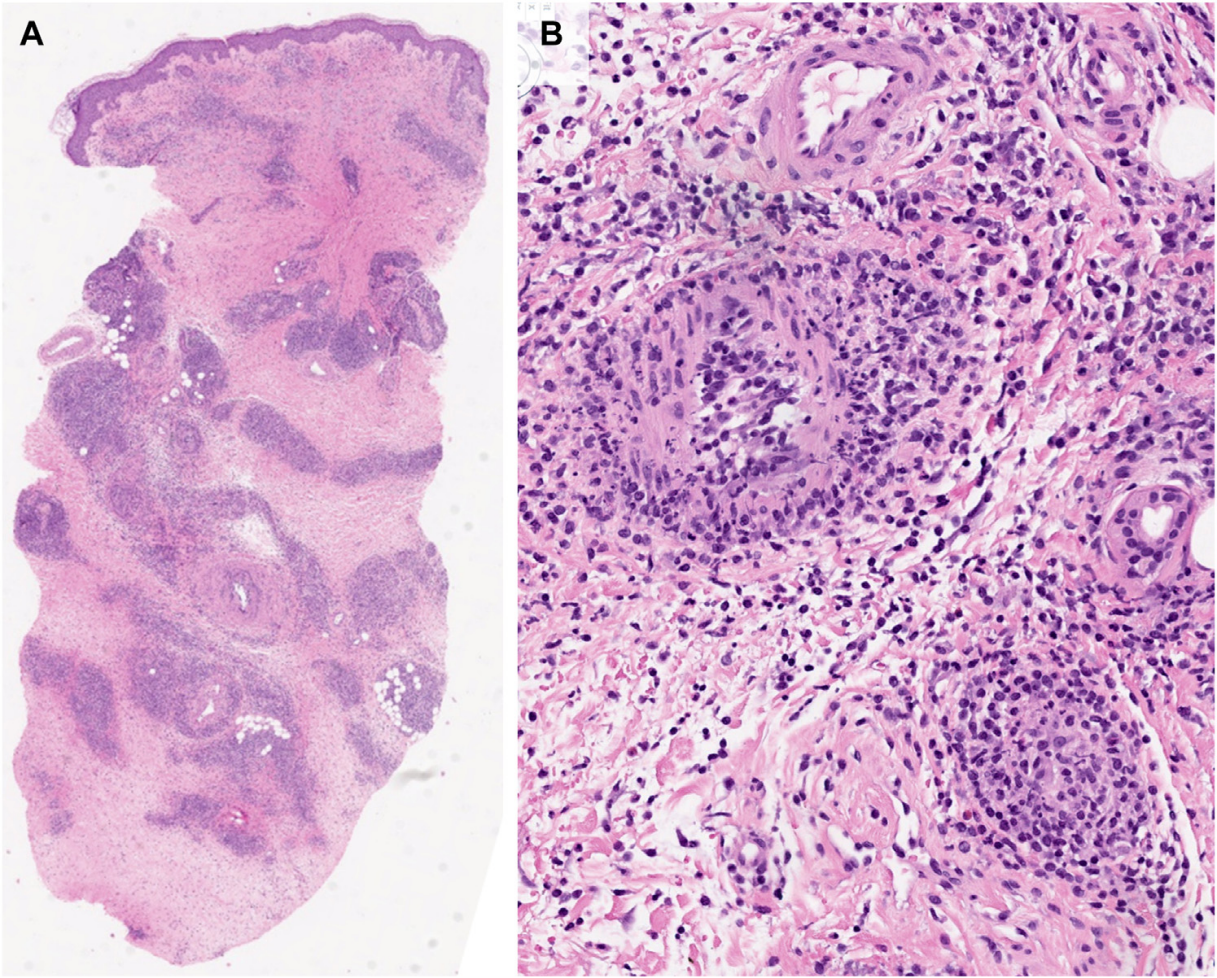
Skin biopsy of the lower leg. H&E stains with (**A**) 4× and (**B**) 100× magnifications, showing dense interstitial and perivascular inflammation involving small and medium-sized blood vessels in the superficial and deep dermis. The inflammatory infiltrate is composed of a predominant population of mononuclear cells with amphophilic cytoplasm mixed with lymphocytes, plasma cells, and histiocytes, and neutrophils with few eosinophils. Neutrophils are seen infiltrating the walls of medium-sized vessels associated with karyorrhexis. There is no prominent fibrinoid necrosis of blood vessel walls.

Skin biopsy revealed dense, interstitial, and perivascular mixed inflammation involving small and medium sized vessels within the superficial and deep dermis, composed of a predominant mononuclear cell population with amphophilic cytoplasm admixed with lymphocytes, plasma cells, histiocytes, scattered neutrophils, and eosinophils with surrounding erythrocyte extravasation and hemosiderin (Fig 3). Non-necrotizing granulomas or interstitial histiocytic clusters were not identified. Immunohistochemical stains demonstrated the predominance of CD68^+^/CD33^+^/lysozyme^+^/CD123^+/−^/CD56^-^/S100^-^/CD1a^-^/TCL1^-^ mononuclear cell population admixed with neutrophils, plasma cells, T-cells, rare B-cells, and histiocytes. IgG4/IgG and CD4/CD8 ratios were normal, with Ki-67 index <2% of the mononuclear infiltrate. Anaplastic lymphoma kinase and human herpesvirus 8 stains were negative. Next generation sequencing was performed on the BM tissue obtained 3 years prior, revealing pertinent mutations in KRAS (G12D), NRAS (G12D), and TET2 (R1261C, R1465∗). Identical genetic alterations, as well as NRAS (G12S) and EZH2 (L149P), were identified by next generation sequencing on the skin biopsy, confirming the diagnosis of LC. Repeat BM biopsy showed a hypercellular marrow at 85% to 90%, blasts <1%, and above NRAS, KRAS, TET2 and EZH2 mutations. The myeloid hyperplasia with increased atypical megakaryocytes was deemed consistent with primary CMML with myelodysplastic syndrome overlap. The patient was started on azacitidine 75 mg/m^2^ for 7 days every 28 days. After 4 cycles of treatment, her skin eruptions did not improve, and repeat bone marrow biopsy showed 95% hypercellularity suggestive of persistent myeloid neoplasm, as well as lambda skewed plasma cells (10% of total marrow cellularity) suspicious for myeloma or a B-cell lymphoproliferative disorder with plasmacytic differentiation. Patient was started on decitabine and venetoclax.

## Discussion

CMML has an incidence of 0.3 per 100,000 people per year in the United States, and most commonly occurs in older adults and males.^2^ The presence of LC in CMML confers poor prognosis and may be a harbinger for progression to AML.^2^ The evolving diagnostic criteria for CMML coupled with the insidious onset of nonspecific clinical findings contributed to the diagnostic delay in this case. The patient’s initial BM biopsy lacked dysplasia which was required for diagnosis of CMML according to the 2016 World Health Organization classification of myeloid neoplasms.^3^ In 2022, the International Consensus Classification of myeloid neoplasms removed dysplasia from the diagnostic criteria.^1^ Current diagnostic criteria includes: monocytosis (≥0.5 × 10^9^/L and ≥ 10% of white blood cell), cytopenia, < 20% blasts in BM and blood, clonality as evidenced by abnormal cytogenetics and/or at least one myeloid associated mutation (with a minimum of 10% allelic frequency), and hypercellular bone marrow that does not meet criteria for AML or other condition with monocytosis.^1^ Over 90% of patients with CMML have a disease-associated genetic mutation, of which approximately 60% involve TET2 and ∼30% RAS genes.^4^ The prognostic and therapeutic significance of these mutations continues to be an area of active research.^4^

Neoplastic cell populations in skin of patients with CMML have been categorized.^5^ In our case without strong evidence of plasmacytoid dendritic cell marker expression, a definite categorization of lesional cells as either myelomonocytic tumors or mature plasmacytoid dendritic cell proliferation is not possible. However, polymorphous skin lesions and histomorphology without prominent blasts and with the background of plasma cells, eosinophils and lymphocytes, align most with CMML with cutaneous involvement by mature plasmacytoid dendritic cell proliferation as previously described.^5^

Correlation of our patient’s BM granulomas with other signs of autoinflammatory disease led to premature closure on a diagnosis of sarcoidosis. Cases of CMML initially misdiagnosed as sarcoidosis have previously been described and may be attributed to shared pathophysiology involving macrophage-associated proliferation of monocytes and other hematopoietic cells.^6^ Approximately 20% of patients with CMML develop an associated systemic inflammatory or autoimmune disease such as vasculitis, arthritis, connective tissue disease and neutrophilic dermatosis.^7^^,^^8^ Leukemic vasculitis, a rare pattern of leukemia cutis, was observed in our patient.^9^ While ocular symptoms are frequently seen with systemic inflammatory or autoimmune diseases, reports of inflammatory eye disease secondary to CMML are limited to isolated case reports.^10^ Uveitis, keratitis, iritis, optic neuropathy, retinal detachment, and choroidal hemorrhage are among the reported patterns of CMML-associated ocular disease.^10^ If left untreated, these processes can lead to irreparable eye damage and permanent vision loss. As skin and eye involvement in the setting of CMML may indicate transformation to AML and carry poor prognosis,^2^ timely and accurate diagnosis is critical.

## Conflicts of interest

None disclosed
